# Predictors of client satisfaction with family planning services in Ethiopia: a systematic review and meta-analysis

**DOI:** 10.3389/fgwh.2024.1430799

**Published:** 2025-01-07

**Authors:** Yeshiwas Ayale Ferede, Worku Chekol Tassew, Agerie Mengistie Zeleke

**Affiliations:** ^1^ Department of Reproductive Health, Teda Health Science College, Gondar, Ethiopia; ^2^ Department of Medical Nursing, Teda Health Science College, Gondar, Ethiopia; ^3^ Department of Clinical Midwifery, Teda Health Science College, Gondar, Ethiopia

**Keywords:** client satisfaction, family planning services, predictors, Ethiopia, systematic review

## Abstract

**Background:**

The use of modern contraceptives by married Ethiopian women has increased over the past 15 years. Despite a few studies reporting different predictors of satisfaction with family planning services, there is a lack of nationwide data showing the determinants of client satisfaction with family planning services. Thus, this meta-analysis aimed to determine the predictors of client satisfaction with family planning services in Ethiopia.

**Methods:**

From January 20 to March 10, 2024, a thorough search of the literature was conducted using PubMed, Web of Science, EMBASE, CINAHL, and Google Scholar. The quality of the included studies was assessed using the critical assessment checklist developed by the Joanna Briggs Institute (JBI). The statistical program Stata 11 was used to carry out the analysis. Using Cochran's Q-statistic, heterogeneity was statistically assessed and measured by the *I*^2^ value. If significant heterogeneity was found across the included studies, a random effects model was used to assess the factors influencing client satisfaction with family planning services. Otherwise, a fixed-effects model was employed.

**Results:**

Independent factors influencing clients' satisfaction with family planning services included waiting times of less than one hour (POR = 4.37; 95% CI: 2.05, 9.32), ensuring privacy (POR = 6.31; 95% CI: 2.78, 14.28), convenient opening hours (POR = 5.91; 95% CI: 1.61, 21.63), education level above primary school (AOR = 2.61; 95% CI: 1.02, 6.68), being informed about side effects (AOR = 3.08; 95% CI: 1.22, 7.74), and receiving adequate information (POR = 4.2; 95% CI: 1.87, 9.44).

**Conclusion:**

The findings indicate that key factors significantly influencing client satisfaction with family planning services include reduced waiting times, privacy protection, convenient service hours, higher education levels, being informed about potential side effects, and receiving comprehensive information. These elements are critical for improving satisfaction and should be prioritized in family planning services. As a result, Ethiopian policymakers and decision-makers must devise plans to maximize client satisfaction with healthcare services through client-centered care.

**Systematic Review Registration:**

https://www.crd.york.ac.uk/prospero/display_record.php?RecordID=563937, PROSPERO (CRD42024563937).

## Background

Family planning is the deliberate choice made by individuals or couples to use natural means and (modern) contraception to determine when to stop having children and how many to have, as well as how to space them (1, 2). In third-world nations, the use of modern family planning services has increased dramatically since the initial programs were launched in the 1950s, when the only available forms of contraception were barrier methods. Family planning, or FP, has since been acknowledged as a fundamental aspect of reproductive health. An enhanced array of options for FP service provision has resulted from increased societal acceptance of family planning (1).

In Africa, especially in sub-Saharan Africa, fecundity, fertility rates, and the unmet need for modern contraceptives are high, where there is still poor usage of contraceptives (2, 3). However, it is now predicted that underdeveloped nations, mostly in Africa, are rapidly adopting modern contraceptive methods. In Eastern Africa, the percentage of women using modern contraceptives is predicted to increase from 43% to 56% between 2017 and 2030. Over 10% of married women worldwide do not use modern contraceptives, meaning that they are fecund and do not have perinatal amenorrhea. These women wish to postpone having children for two years or longer or to stop having children altogether, but they do not use modern contraceptives (2, 4). Twenty percent of married women in Africa lack access to modern contraception. On the other hand, the UN (United States) estimates that the number of women worldwide who do not have access to modern contraception will decrease to 139 million in 2030 from 142 million in 2017. With a projected reduction from 22% in 2017% to 16% in 2030, Eastern Africa is predicted to experience the greatest decrease (5).

Even though the use of modern contraceptives by married Ethiopian women has increased over the past 15 years, increasing from 6.9 to 13.9, 21, and 40% in 2000, 2005, 2011, and 2016, respectively. According to the 2016 EDHS, 55% of the single, sexually active women in Ethiopia used modern FP methods (5, 6). However, there is still a high unmet need for contraceptives in Ethiopia.

It is evident that FPs are essential to women's and families' health. It also significantly affects the social and economic circumstances of families. It enhances socioeconomic conditions in the community, reduces poverty, empowers women, and enhances biological, social, mental, and psychological health (1, 6). In light of this, the FP2020 recognizes and advocates for the right of all FP users to the best possible service; moreover, the data indicate that inadequate service delivery is the primary barrier to the adoption and ongoing use of the modern FP globally. Further study is necessary to meet client needs and implement appropriate solutions. Increasing the number of new FP users and decreasing discontinuation both depend on ensuring client happiness (3).

It has been shown that a client's perception of continuity of care and client satisfaction are correlated (7). It is seen as an indicator of high-quality service delivery (8–10). Client satisfaction is a relative concept that takes into account clients' expectations of the healthcare system, perceived needs, and overall health care experience (11, 12). According to some studies, a client's decision to utilize services, to keep using them, or to return for more services is largely influenced by their level of satisfaction (7). As a recognized component of care quality, assessing the determinants of satisfaction offers a great chance to include clients in the process of evaluating programs from the viewpoint of users (13, 14).

Several studies have demonstrated that a variety of factors influence client satisfaction. These factors include the following: sociodemographic factors (age, occupation, and education of the client) (10, 12); health facility factors (clinic cleanliness, ease of access, hours of operation, and wait times) (10, 12, 13, 15, 16); interpersonal factors (poor obstetric history, attitude and knowledge, and experiencing side effects) (16, 17); and client-provider factors (privacy and information provided) (12, 18–20).

Although the same review was previously conducted in Ethiopia, it did not address the critical factors influencing client satisfaction with family planning services. Consequently, by incorporating new research and offering a more thorough understanding of the factors influencing satisfaction, repeated reviews can help close these gaps (21).

Although understanding the factors affecting client satisfaction is crucial, previous reviews in Ethiopia have not fully explored these determinants. This meta-analysis addresses this gap by aggregating data from various studies to offer a thorough insight into what influences client satisfaction with family planning services in Ethiopia. By pinpointing these key factors, the study aims to inform the development of targeted interventions, guide policy changes, and enhance service quality. The findings are significant for improving family planning services, as they provide direction for policy development, service enhancements, and provider training, while also emphasizing the importance of continuous monitoring to better meet client needs and increase service accessibility across the country.

## Methods

Preferred Reporting Items for Systematic Reviews and Meta-Analyses (PRISMA) reporting criteria were followed for the conduct and reporting of the study (22) Supplementary File S1. The review was registered with the International Prospective Register of Systematic Reviews (PROSPERO) under the unique identifier CRD42024563937.

### The eligibility criteria

Studies involving Ethiopian women who were sexually active and of childbearing age were considered. Included were articles that provided the binary factors associated with women's use of family planning services as well as the odds ratio (OR) and 95% confidence interval (CI). Included were studies that were written in or published in English but were only performed in Ethiopia. Conversely, research that omitted any discussion of factors affecting clients' satisfaction with family planning services was excluded. Letters, reviews, commentaries, abstracts, editorial reports, and reviews were also not included in the study.

### Information sources and search strategy

A systematic literature search was performed on PubMed, Web of Science, EMBASE, CINAHL, and Google Scholar from January 20 to March 10, 2024. Medical subject headings, keywords, different Boolean operators, and truncations were used in the search strategy. The following search keywords were used: “client satisfaction” OR “women satisfaction” AND “family planning services” OR “contraceptive services” AND Determinant* OR Predictor* AND “Ethiopia”. Furthermore, the bibliographies of selected articles were reviewed for additional potentially relevant studies.

### Outcome

The primary findings of this meta-analysis were factors that predict Ethiopian clients' satisfaction with family planning services. Customer satisfaction with FP services and staff is a measure of the quality of care provided (23). Every woman in the meta-analysis who was of reproductive age had used family planning services at some point (24).

### Study selection

The citation manager program EndNote X7 was utilized to retrieve all articles and oversee the screening procedure. According to their titles and abstracts, the studies were first evaluated. After that, full-text reviews of the studies that were found to be pertinent were obtained. To describe the study selection processes, the PRISMA 2020 flow diagram for new systematic reviews was used.

### Data extraction

YAF and AMZ, the two reviewers, used Microsoft Excel to separately extract relevant data from each included article. We extracted the first author, publication year, study design and setting, sample size, and various determinants of family planning satisfaction from the included articles.

### Risk of bias

Two reviewers (YAF and WCT) independently assessed the quality of the included papers using the Joanna Briggs Institute (JBI) critical appraisal checklist, which was modified for case‒control and cross-sectional research. Disagreements or confusing information between the two reviewers were resolved through discussion. Case‒control studies were scored on a zero‒ten-point scale based on factors such as statistical analysis, standard measurement, group matching and comparability, cofounder management techniques, and standard measurement. Cross-sectional studies were rated on a zero–eight point scale according to how well they addressed the target population, the suitability of the sample size, the data collection techniques, the definition of the variables, the data collection instruments, the statistical analysis tests, the study objectives, and the quality of the response rate. A low risk score is five or above.

### Data synthesis

The odds ratio (OR) and 95% confidence interval (CI) for each factor were calculated for each included study. YAF and WCT, two reviewers, carried out the data synthesis. With Stata 11 statistical software, we calculated the effects of summary estimates using the “metan” command. Using Cochran's Q-statistic, heterogeneity was statistically assessed and measured by the *I*^2^ value. A random-effects model was used to estimate the determinants of client satisfaction with family planning services due to the existence of heterogeneity across the included studies. Visual inspection of asymmetry in funnel plots and Egger regression tests with a *p* value less than 0.05 were used as cutoff points to determine the presence of publication bias.

## Results

### Study selection

**Figure 1:**
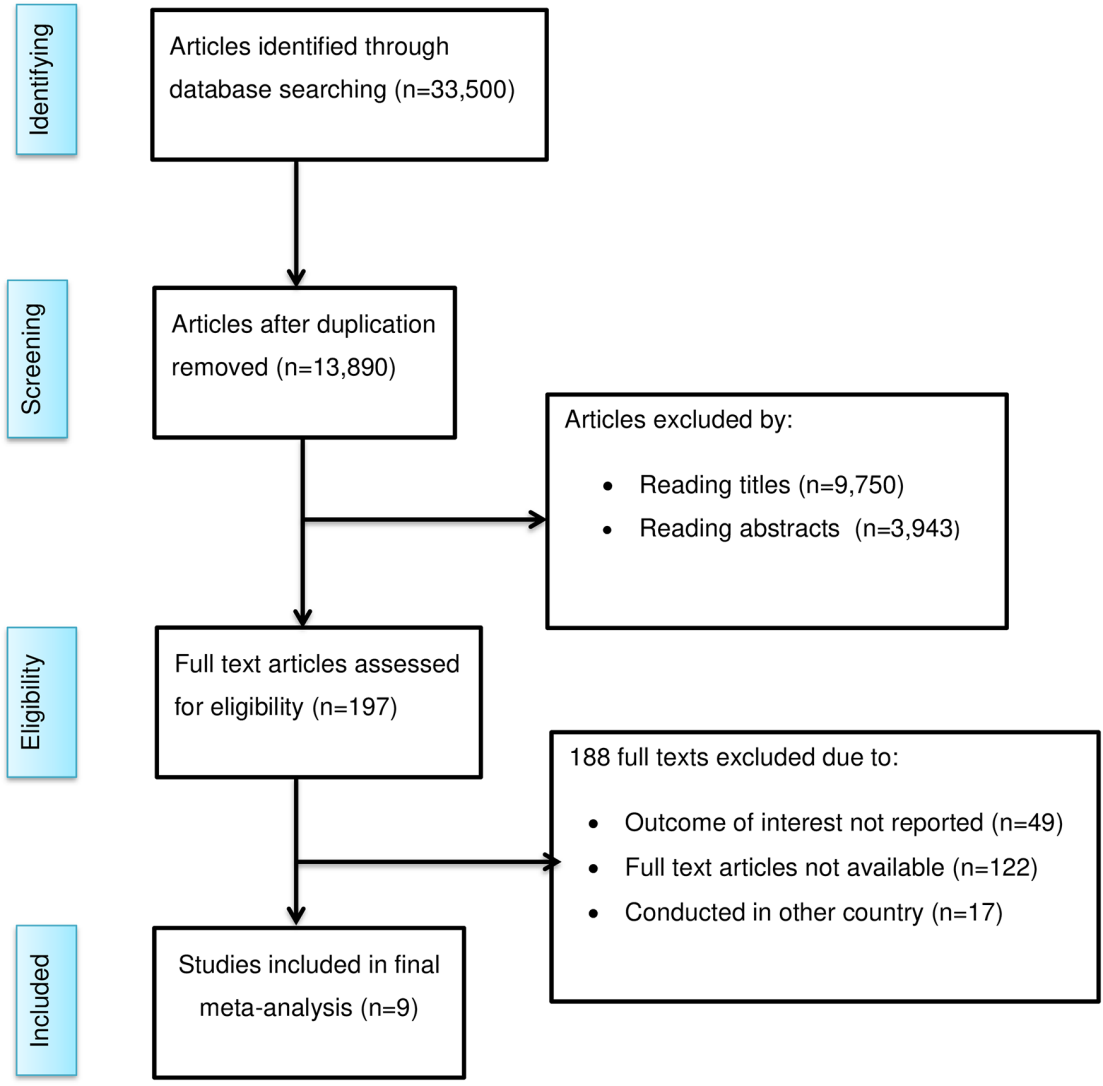
PRISMA flow diagram of study selection for the meta-analysis of predictors of client satisfaction with family planning services in Ethiopia, 2024.

A total of 33,500 documents were identified using various databases. Approximately 19,610 duplicate records were removed before screening. After independent evaluation of 13,890 records by two reviewers (YAF and AMZ) based on their titles and abstracts, 13,693 records were further excluded. After reviewing 197 full-text articles, an additional 188 articles were removed. Ultimately, nine studies were included in the final analysis (Figure 1).

### Study characteristics

All of the studies that were included were cross-sectional. The included studies had sample sizes ranging from 278 to 538. This cross-sectional study scored seven to eight on the Joanna Briggs Institute Critical Appraisal Checklist. A total of 3,872 women who used family planning services were included. In terms of regional distribution, five studies were conducted in the southern region (25–28), two in Oromia (24, 29), one in Somalia (7), and one in Amhara (30) (Table 1).

**Table 1:** Summary of studies included in the meta-analysis of predictors of client satisfaction with family planning in Ethiopia, 2024.

Id	Author	Pub year	Region	Study area	Study design	Sample size
1	Asrat et al. (31)	2018	Amhara	BahirDar	Cross-sectional	491
2	Argagol et al. (28)	2015	Southern	Hossana	Cross-sectional	334
3	Dulla et al. (27)	2019	Southern	Kucha	Cross-sectional	512
4	Gebreyesus (7)	2019	Somalia	Jijiga	Cross-sectional	492
5	Wogu et al. (26)	2020	Southern	Tembaro	Cross-sectional	411
6	Hunduma J et al. (32)	2018	Southern	Sodo	Cross-sectional	421
7	Beyene et al. (24)	2022	Oromia	Adama	Cross-sectional	417
8	Anne et al. (25)	2021	Southern	Halaba	Cross-sectional	538
9	Bezawit B. et al. (29)	2019	Oromia	Jima	Cross-sectional	278

### Meta-analysis

#### Risk of bias assessment for the included studies

The critical evaluation checklist was developed by the Joanna Briggs Institute (JBI) and adjusted for cross-sectional studies. The quality evaluation summary showed that more than three-fourths (*n* = 7, 77.7%) of the included studies were of high quality, while the remaining one-fifth (*n* = 2, 22.2%) of the studies were of medium quality (Supplementary File S2).

**Figure 2:**
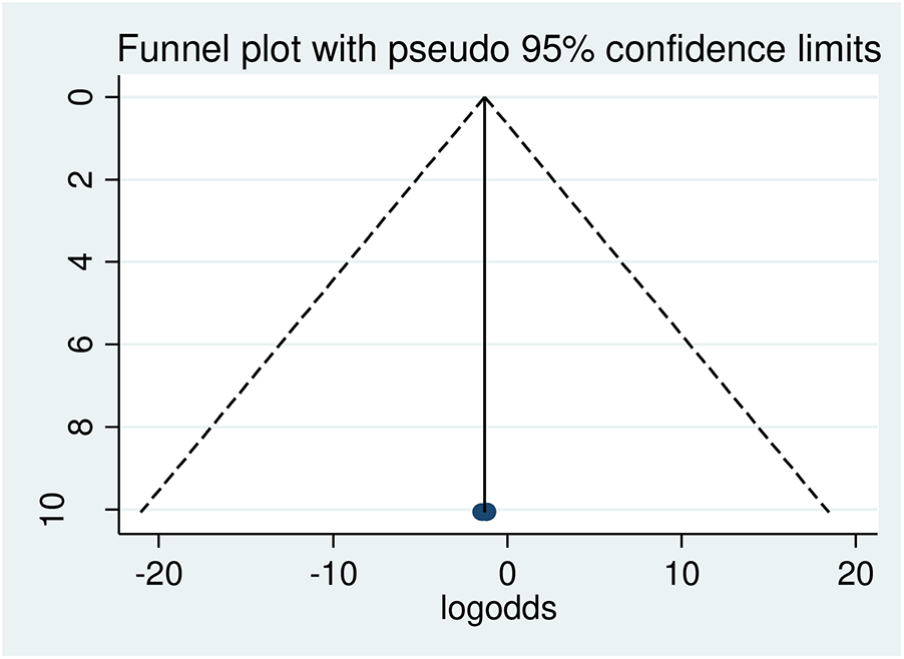
Graphic representation of publication bias using funnel plot, 2024.

Publication bias: To determine whether publication bias existed, a visual examination of the asymmetry in funnel plots and Egger regression tests were used. As a consequence, the results of Egger's tests and funnel plots indicated that publication bias existed in the included papers. Egger's test revealed the existence of publication bias, with a statistically significant result (*p* = 0.000). Additionally, an examination of the funnel plots revealed an uneven distribution of the studies (Figure 2).

**Figure 3:**
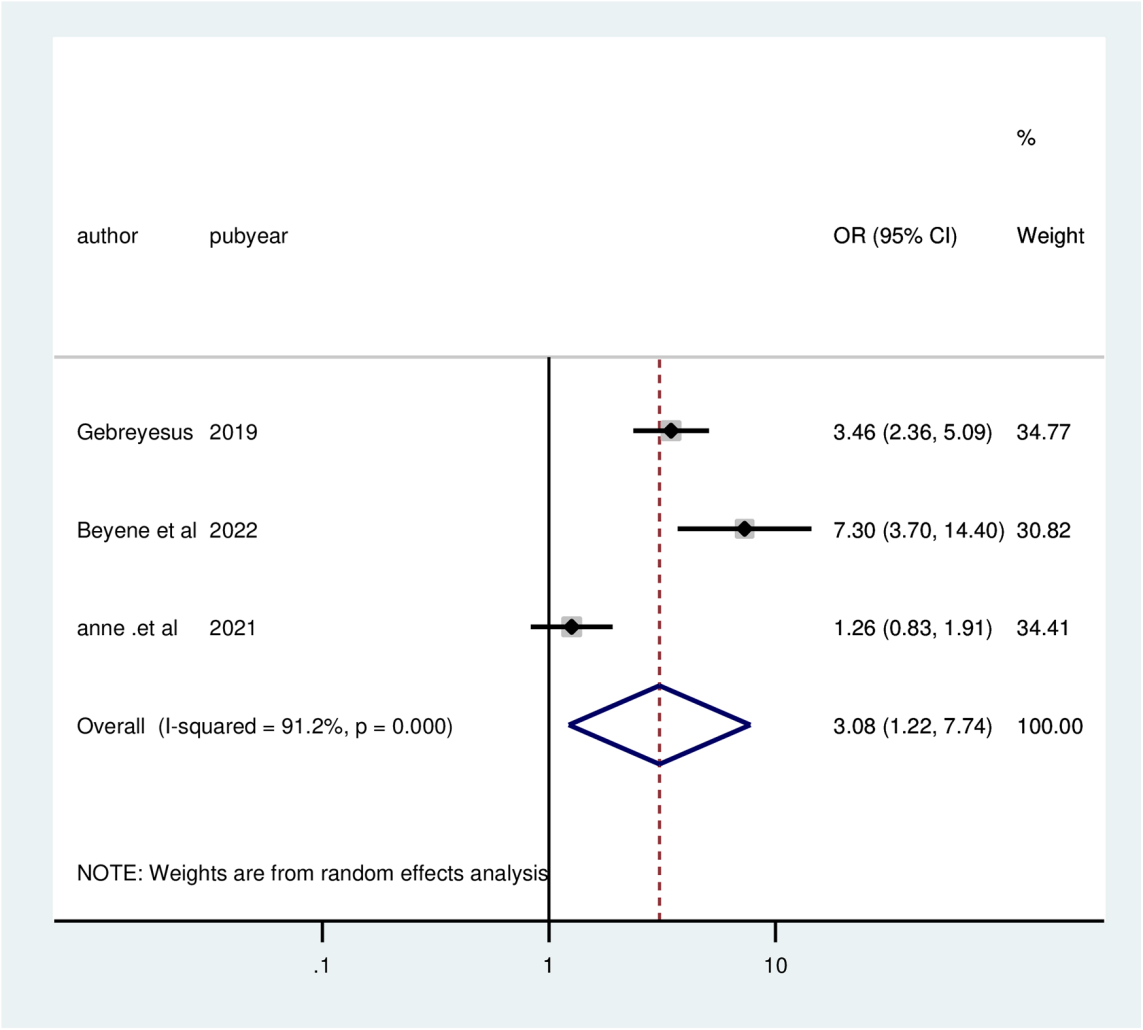
Shows output of sensitivity analysis of included studies, 2024.

Sensitivity analysis: Because there was publication bias, a sensitivity analysis was performed. The sensitivity analysis revealed no significant studies (Figure 3).

#### Determinants of client satisfaction with family planning services

Due to the uneven classification (grouping) of the independent variables about the outcome, some of the factors associated with client satisfaction with family planning services were quantitatively pooled, while others were not. According to this study, waiting times, privacy, opening hours, and adequate information were significantly associated with client satisfaction, whereas educational level and explaining side effects were not significantly associated.

**Figure 4:**
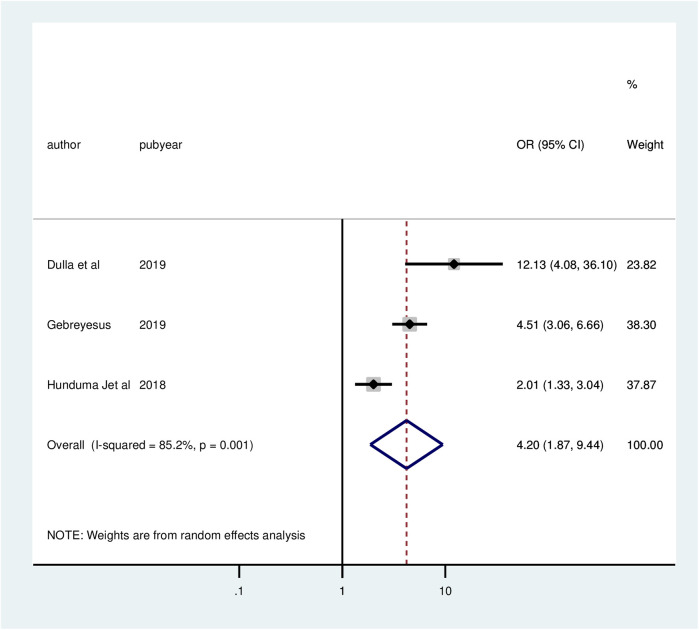
Forest plot of the association between waiting time and client satisfaction with family planning services, 2024.

According to seven studies, client satisfaction with family planning services is significantly correlated with waiting times. When comparing customers with waiting times under thirty minutes to those with waiting times above thirty minutes, the chances of client satisfaction were 4.37 times (AOR = 4.37; 95% CI: 2.05, 9.32) greater for those with shorter wait times. The meta-analysis revealed significant heterogeneity among the included studies (*I*^2^ = 91.9%, *P* = 0.000). Thus, a random effect model analysis was used (Figure 4).

**Figure 5:**
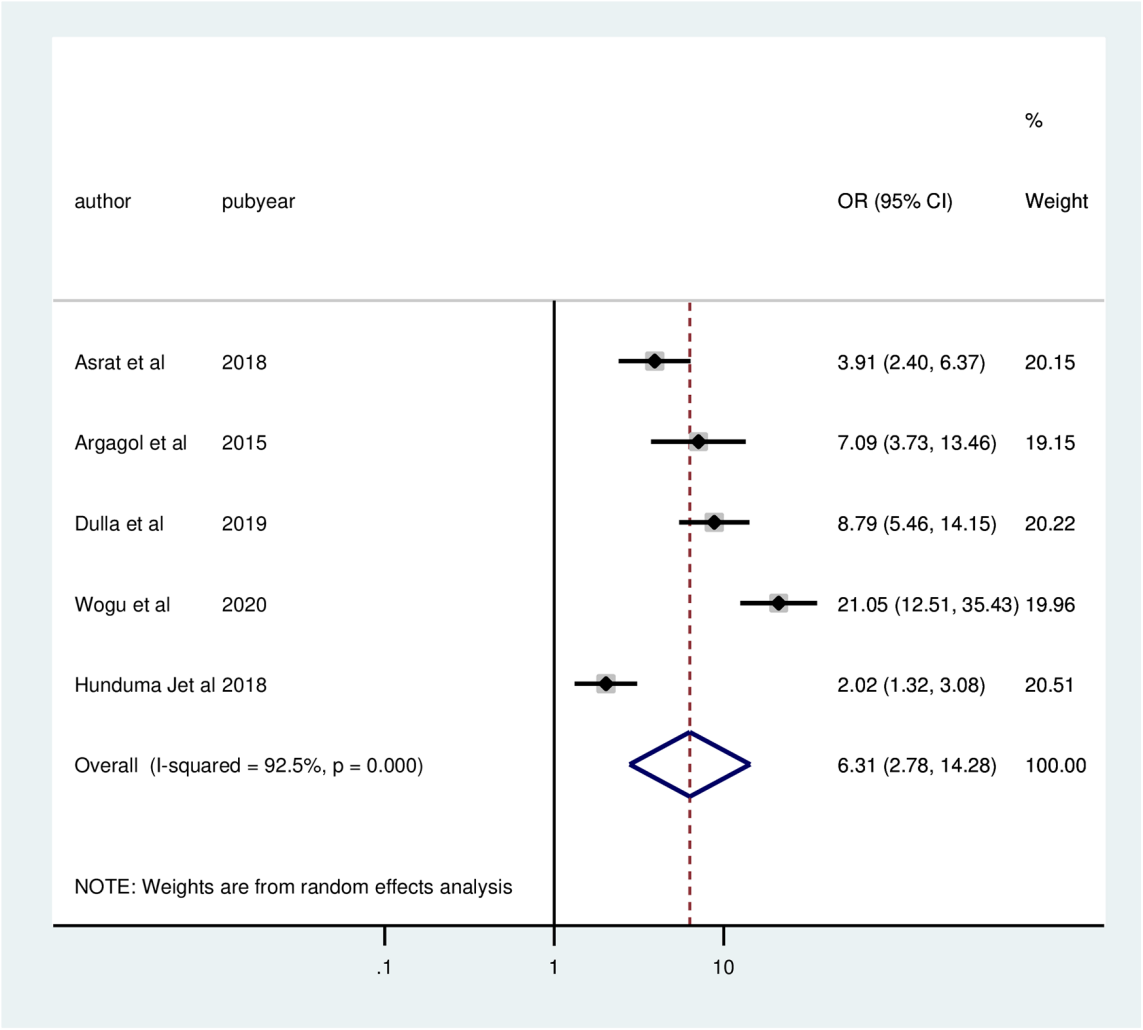
Forest plot of the association between privacy and client satisfaction with family planning services, 2024.

Five studies revealed a significant association between client satisfaction with family planning services and privacy. Compared to clients whose privacy was not preserved, the chances of client satisfaction were 6.31 times greater (AOR = 6.31; 95% CI: 2.78, 14.28) for clients whose privacy was maintained. The meta-analysis revealed significant heterogeneity among the included studies (*I*^2^ = 92.5%, *P* = 0.000). Thus, a random effect model analysis was used (Figure 5).

**Figure 6:**
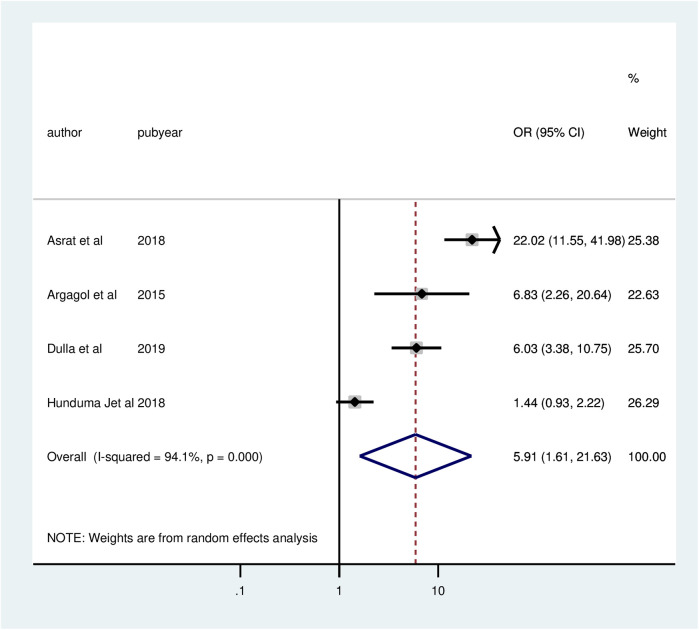
Forest plot of the association between opening hour and client satisfaction with family planning services, 2024.

Four studies found a substantial correlation between customer satisfaction with family planning services and opening hours. The chances of client satisfaction were 5.91 times greater for clients who did not open a convenient clinic (AOR = 5.91; 95% CI: 1.61, 21.63) than for those who did not. The meta-analysis revealed significant heterogeneity among the included studies (*I*^2^ = 94.1%, *P* = 0.000). Thus, a random effect model analysis was used (Figure 6).

**Figure 7:**
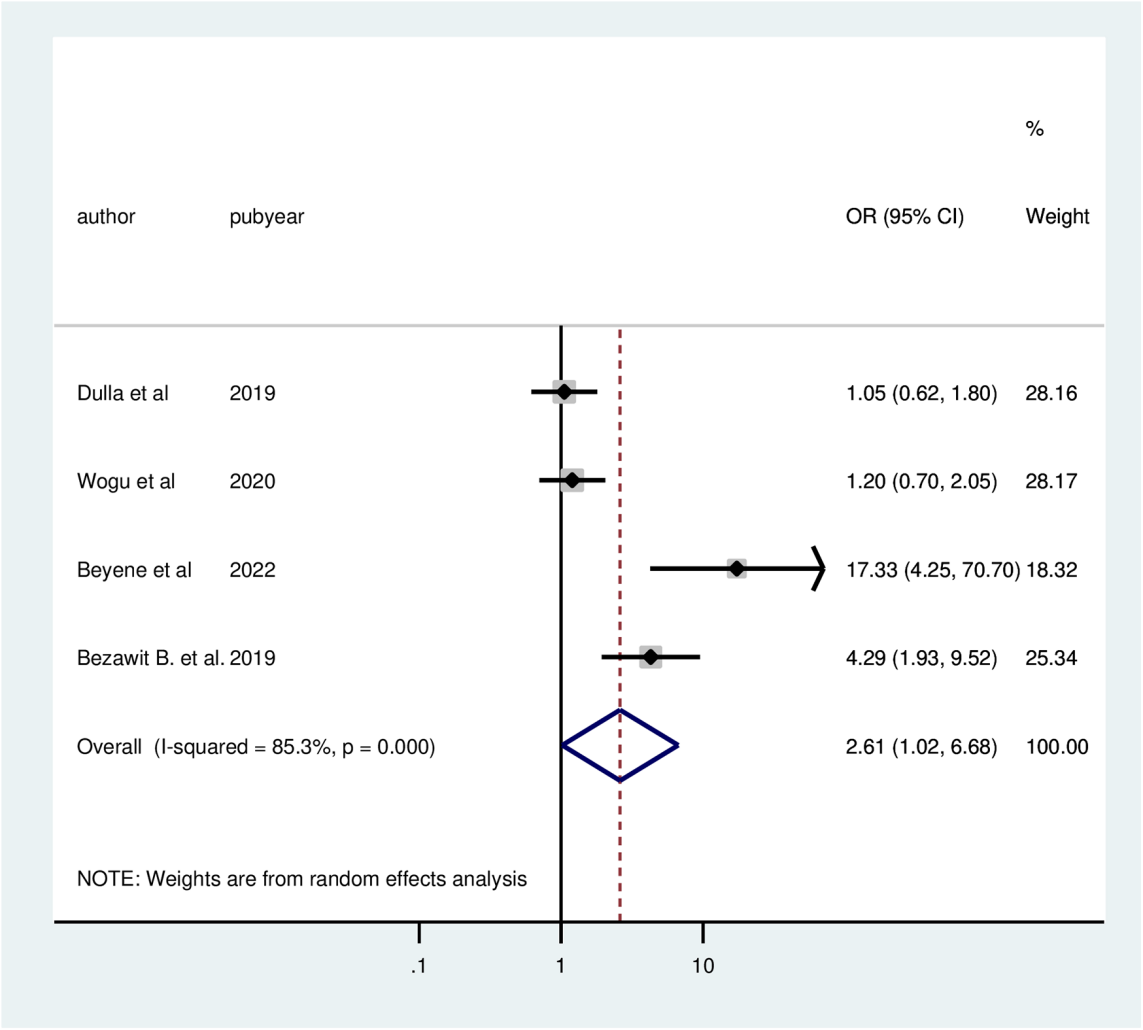
Forest plot of the association between information and client satisfaction with family planning services, 2024.

Three studies indicated that information has a significant association with client satisfaction in family planning services. The odds of client satisfaction were 4.2 times greater (AOR = 4.2; 95% CI: 1.87, 9.44) among clients who received adequate information than among those who did not receive adequate information. In this meta-analysis, the included studies exhibited substantial heterogeneity (*I*^2^ = 85.2%, *P* = 0.001). Thus, a random effect model analysis was used (Figure 7).

**Figure 8:**
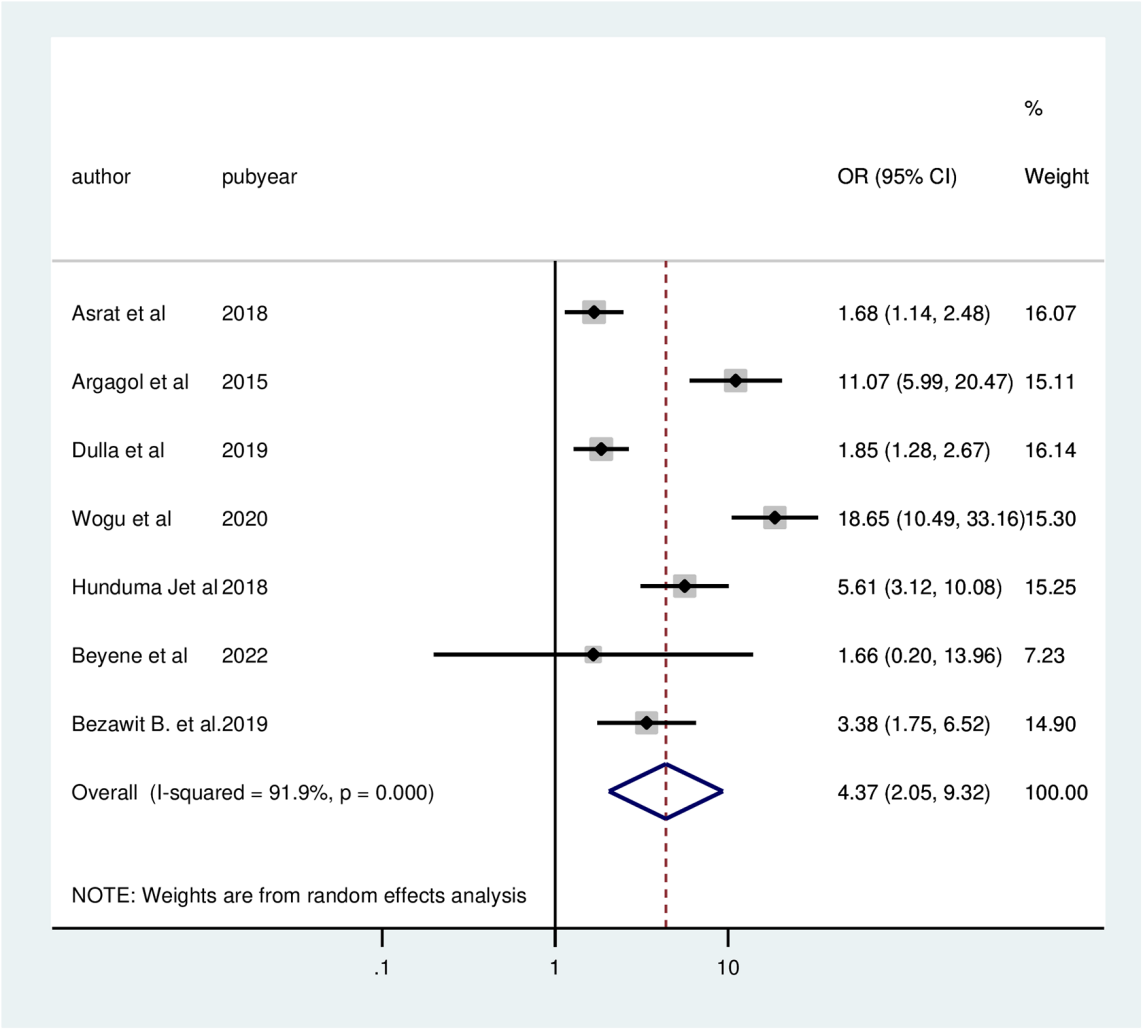
Forest plot of the association between educational level and client satisfaction with family planning services, 2024.

Four studies demonstrated a significant correlation between respondents' educational levels and client satisfaction with family planning services. Clients with education levels above primary school had a 2.61 times higher likelihood of satisfaction (AOR = 2.61; 95% CI: 1.02, 6.68) compared to those with education below secondary school. Due to considerable heterogeneity among the included studies (*I*^2^ = 85.3%, *P* = 0.000), a random effects model was utilized for the analysis (Figure 8).

**Figure 9:**
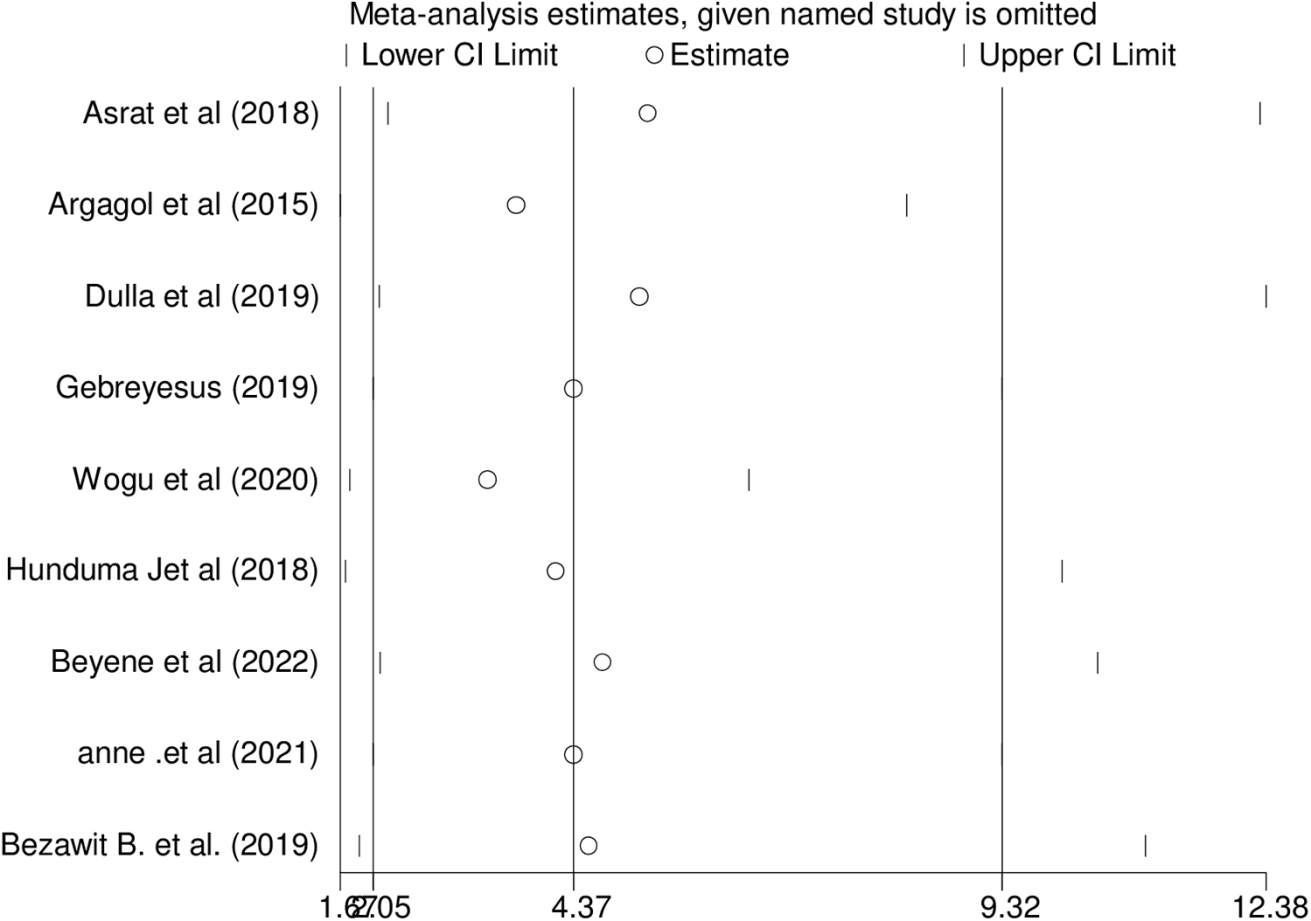
Forest plot of the association between explain side effect and client satisfaction with family planning services, 2024.

Three studies found a significant relationship between the clear explanation of side effects and client satisfaction in family planning services. Clients who received clear explanations of side effects were 3.08 times more likely to be satisfied (AOR = 3.08; 95% CI: 1.22, 7.74) compared to those who did not. Due to significant heterogeneity among the studies (*I*^2^ = 91.2%, *P* = 0.000), a random effects model was utilized for the analysis (Figure 9).

## Discussion

Patient satisfaction is indeed an important factor in assessing the quality of care, particularly in healthcare settings catering to women (32). In this meta-analysis, the factors influencing female family planning users' satisfaction with their services were investigated. Accordingly, a waiting time of less than half an hour, a convenient opening hour, ensuring privacy, and receiving adequate information were significant determinants of client satisfaction with family planning services. Therefore, understanding and addressing the factors that influence satisfaction with family planning services is essential for promoting their effective use. This can have a significant impact on individual well-being and contribute to achieving broader public health goals.

In this study, the main criterion impacting satisfaction was determined to be the sufficiency of the information provided. Compared to mothers who did not receive enough information, mothers who did receive enough information were more satisfied. This finding is also consistent with findings from a study performed in Nigeria, which indicated that patients who received respectful and deferential treatment from other clinic staff members were twice as likely to be satisfied (15).

In this study, service waiting time was also found to be one of the factors that affect family planning service satisfaction. Clients with a short waiting time were more likely to be satisfied than those with a long waiting time, and this evidence was supported by other studies (33, 34). The possible reason might be Clients often perceive shorter waits as a sign of efficient and well-organized services, enhancing their trust in the facility and its staff. Quick service delivery also minimizes the risk of clients leaving without receiving care or second-guessing their decisions, while allowing more time for meaningful provider-client interactions. Additionally, reduced waiting times encourage repeat visits, improving service utilization and long-term satisfaction.

Additionally, our study revealed that when respondents felt that their privacy was protected, they were more satisfied with FP services. This finding is consistent with a study from Mozambique, where women's satisfaction with FP service was found to be strongly influenced by poor healthcare provider-client contact (35). Similarly, findings from Mexico suggest a direct link between uninterrupted consultations and increased client satisfaction (36). A possible explanation for this might be that family planning is a very private matter, and many people find it difficult to talk about their issues in public. For this reason, maintaining anonymity is crucial while offering family planning services. Previous studies have demonstrated that clients feel more at ease when clinicians respect their privacy during counseling sessions and tests (37).

Moreover, this study revealed that the number of family planning service hours is one of the factors that influence client satisfaction with the service; clients who had convenient service hours were more likely to be satisfied with the family planning service than were those who were not. A possible reason might be that women will be better able to use the service if it is available at a convenient time; thus, family planning services are provided without interfering with office work hours. This evidence is supported by previous studies (38).

Furthermore, respondents with an educational level higher than primary school were more likely to express satisfaction with family planning (FP) service delivery compared to those with less than a primary school education. One potential reason for this could be that higher education often equips individuals with better access to information regarding FP methods and services, enabling them to make well-informed decisions and set realistic expectations about the services they receive (39).

Lastly, our findings indicate that respondents who were clearly informed about the side effects of contraceptives reported higher levels of satisfaction with FP services compared to those who did not receive this information. When individuals are well-informed about potential side effects, they can make decisions that better align with their personal preferences and health needs, reducing the likelihood of unexpected outcomes and thus increasing their satisfaction with the services (40).

## Strength and limitations of the study

This study aims to deliver a more comprehensive and robust synthesis by incorporating a broader array of studies, thus providing a more current evaluation of client satisfaction than previous reviews. While many earlier reviews concentrated on overall satisfaction levels without exploring specific predictors, this research distinguishes itself by identifying and quantifying the key factors that influence client satisfaction. Moreover, to the authors' knowledge, this meta-analysis is the first to quantitatively assess the variables affecting client satisfaction with family planning services in Ethiopia.

This review has the following potential limitations: First, the possible sources of heterogeneity were not thoroughly addressed, despite the presence of heterogeneity between the included studies. Additionally, the studies included in this review were limited to only four regions of the country, which may affect the generalizability of the findings.

To help policymakers and other pertinent stakeholders understand the impact of satisfaction on the use of continuous family planning services, this meta-analysis offers crucial evidence. Several factors have been found to be associated with a greater likelihood of client satisfaction with family planning services. Consequently, it is preferable to focus on maintaining privacy during counseling for family planning services, reducing service waiting times, receiving sufficient information, educational level above primary school, clear explanation about side effect, and enabling convenient service hours for family planning service delivery to increase client satisfaction with family planning services in Ethiopia.

## Conclusion

The findings of this meta-analysis indicate that several factors significantly influence client satisfaction with family planning services, including wait times of less than half an hour, convenient clinic hours, privacy assurance, clients' education level beyond primary school, clear explanation of side effects, and provision of adequate information. To boost client satisfaction in family planning services, reduce wait times, offer flexible clinic hours, and ensure privacy. Enhance education by providing clear, understandable information and train providers to communicate side effects effectively. Focus on client-centered care through continuous staff training. Implement feedback mechanisms for ongoing improvements.

## Data Availability

The original contributions presented in the study are included in the article/Supplementary Material, further inquiries can be directed to the corresponding author.
