# Supplementary material for: Predictors of client satisfaction with family planning services in Ethiopia: a systematic review and meta-analysis
Source: Front Glob Womens Health. 2025 Jan 7;5:1430799. doi: 10.3389/fgwh.2024.1430799 (PMC11747235; doi:10.3389/fgwh.2024.1430799)
Supplement: Supplementary file 2 [file Table2.docx]

Table 2: Quality assessment of included articles using Joanna Briggs Institute (JBI) critical appraisal checklist

| Corresponding author |  | Criteria | | | | | | | |  |  |
| --- | --- | --- | --- | --- | --- | --- | --- | --- | --- | --- | --- |
|  | Study Design | Sample frame appropriateness | Sampled appropriately | SS | Describe subjects | Analysis | Validity | Reliability | Statistical test | RR | Total |
| Asrat et al | IBCS | 1 | 1 | 0 | 1 | 1 | 1 | 1 | 1 | 1 | 8 |
| Argagol et al | IBCS | 1 | 1 | 0 | 1 | 1 | 1 | 1 | 1 | 1 | 8 |
| Dulla et al | IBCS | 0 | 1 | 0 | 1 | 1 | 1 | 1 | 1 | 1 | 7 |
| Gebreyesus | IBCS | 0 | 1 | 0 | 1 | 1 | 1 | 1 | 1 | 0 | 6 |
| Wogu et al | IBCS | 1 | 1 | 0 | 1 | 1 | 1 | 1 | 1 | 1 | 8 |
| Hunduma Jet al | IBCS | 1 | 1 | 1 | 1 | 1 | 1 | 1 | 0 | 1 | 8 |
| Beyene et al | IBCS | 1 | 1 | 0 | 1 | 1 | 0 | 0 | 1 | 1 | 6 |
| Anne .et al | IBCS | 1 | 1 | 1 | 1 | 1 | 1 | 1 | 1 | 1 | 9 |
| Bezawit B. et al. | IBCS | 1 | 1 | 0 | 1 | 1 | 1 | 1 | 1 | 1 | 8 |

** IBCS, Institutional Based Cross-sectional Study, SS, Sample Size, RR, Response Rate
